# Influence of a dynamic rearing environment on development of metabolic phenotypes in age-0 Lake Sturgeon, *Acipenser fulvescens*

**DOI:** 10.1093/conphys/coz055

**Published:** 2019-10-11

**Authors:** Gwangseok R Yoon, David Deslauriers, W Gary Anderson

**Affiliations:** 1 Department of Biological Sciences, University of Manitoba, Winnipeg, MB R3T 2N2, Canada; 2 Freshwater Institute, Fisheries and Oceans Canada, Winnipeg, MB R3T 2N6, Canada

**Keywords:** Conservation aquaculture, environmental matching hypothesis, lake sturgeon, metabolic phenotypes

## Abstract

Environment–phenotype interactions are the most pronounced during early life stages and can strongly influence metabolism and ultimately ecological fitness. In the present study, we examined the effect of temperature [ambient river temperature (ART) vs ART+2°C], dissolved oxygen (DO; 100% vs 80%) and substrate (presence vs absence) on standard metabolic rate, forced maximum metabolic rate and metabolic scope with Fulton’s condition factor (K), energy density (ED) and critical thermal maximum (CTmax) in age-0 Lake Sturgeon, *Acipenser fulvescens*, before and after a simulated overwintering event. We found that all the environmental variables strongly influenced survival, K, ED and CTmax. Fish reared in elevated temperature showed higher mortality and reduced K pre-winter at 127 days post-hatch (dph). Interestingly, we did not find any significant difference in terms of metabolic rate between treatments at both sampling points of pre- and post-winter. Long-term exposure to 80% DO reduced ED in Lake Sturgeon post-winter at 272 dph. Our data suggest that substrate should be removed at the onset of exogenous feeding to enhance the survival rate of age-0 Lake Sturgeon in the first year of life. Effects of early rearing environment during larval development on survival over winter are discussed with respect to successful recruitment of stock enhanced Lake Sturgeon, a species that is at risk throughout its natural range.

## Introduction

Early phenotypic development is known to strongly influence growth trajectory and ecological fitness of individuals (Lindström, 1999). Thus, the importance of the environment during early development has been well studied across taxa, and a number of models have been proposed to understand the long-term consequences (Monaghan, 2008). The environmental matching hypothesis suggests that thrifty phenotypes proliferate in a suboptimal environment, but they may become maladaptive when the environment becomes mismatched (Monaghan, 2008). When the mismatch is negligible, individual phenotypic plasticity should permit adaptation to the new environment; however, when the mismatch is greater than their adaptive capability, individuals may show reduced fitness in the long term (Hendry *et al*., 2011).

The environmental matching hypothesis has many implications for conservation biology in instances where captive-reared individuals are released into the wild to enhance the status of endangered or at-risk populations. Conservation fish hatcheries typically employ artificial rearing environments to facilitate hatchery management, but such environments lack the variability found in the wild and often result in lower phenotypic variation in progeny (Johnsson *et al*., 2014). For instance, absence of complexity in rearing environments can result in naïve individuals towards natural selective pressures (Araki *et al*., 2008). Practically it is impossible to implement all wild environmental variables in a hatchery, thus, understanding key environmental variables at critical life stages is important to produce more suitable individuals that are likely to increase post-release survival rates (Roberts *et al*., 2011, 2014). Environmental enrichment in artificial settings has been used to mimic key factors from the natural environment to naturalize phenotypes and promote acquisition of vital skills as well as growth performance (McAdam, 2011; Lönnstedt *et al*., 2012; Chivers and Ferrari, 2013). For example, previous studies have indicated that simple inclusion of substrate (e.g. gravel) at the appropriate time can promote growth performance in sturgeon species (Gessner *et al*., 2009; Bates *et al*., 2014). Thus, understanding how key abiotic factors shape phenotypic development and how these phenotypes will influence survival rates in the first year of life, particularly during the challenging first overwintering event, would directly benefit conservation programs (Johnsson *et al.*, 2014).

Environmental temperature is the key to biological phenomena as it dictates the rate of biochemical reactions and strongly influences physiological processes, locomotion and the geographical distribution of many fish species (Schulte *et al*., 2011; Stewart and Allen, 2014). Standard metabolic rate (SMR) is defined as the minimum oxygen consumption rate to maintain vital processes such as cellular homeostasis and organismal integrity (Hochachka and Somero, 2001; Treberg *et al*., 2016). SMR in fish may play an important role in ecological fitness because SMR is known to be correlated with growth trajectories, maximum aerobic performance and some behaviours (Frappell and Butler, 2004; Burton *et al*., 2011; Norin and Clark, 2016). Maximum metabolic rate (MMR) is the maximum oxygen consumption rate required to maintain maximum activity. MMR is important in prey capture, predator escape, reproduction and habitat use as all these ecological factors are largely dependent on the fish’s swimming ability (Kieffer, 2000). The difference between SMR and MMR is defined as metabolic scope (MS), which is the aerobic capacity for routine activities such as foraging, growth, locomotion and reproduction (Hochachka and Somero, 2001). The relationship between MS and temperature has been described by a unimodal curve with the upper limit known as critical thermal maximum (CTmax) beyond which normal physiological function cannot meet the oxygen demand of tissues thus leading to metabolic suppression (Pörtner, 2010). Specifically, decreased MS due to increased temperature and hypoxia can be detrimental for fish because of the importance of MS for growth, reproduction and ecological fitness (Pörtner, 2010; Holt and Jørgensen, 2015).

Warmer aquatic environments decrease the solubility of oxygen, further, increased temperature results in an increased metabolic demand for most ectotherms leading to increased oxygen consumption rates. Studies have suggested that increased temperature and hypoxia may have a synergistic effect (Pörtner, 2010; McBryan *et al*., 2013) as temperature increases metabolic rate in fish (Clarke and Johnston, 1999; Jo and Kim, 1999) and hypoxia can negatively impact locomotion, growth, development of the embryo and aerobic metabolism coupled with increased levels of glycolysis (Baker *et al*., 2005; Behrens and Steffensen, 2007; Farrell and Richards, 2009; Richards, 2009; Dupont-Prinet *et al*., 2013). Previous results have indicated that acute exposure to hypoxia down to 80% of air saturation did not affect aerobic metabolism or hypoxia avoidance behaviours in a number of species of fish (Poulsen *et al*., 2011; McBryan *et al*., 2013; Svendsen *et al*., 2014); however, long-term exposure to 80% dissolved oxygen (DO) could result in increased mortality and loss of aerobic capacity following exposure to a stressful environment (i.e. low temperature and food deprivation; Yoon *et al*., 2018). Previous studies have predominantly focused on teleost fish (Fernandes *et al*., 1995; Hobbs and Mcdonald, 2010), but these relationships remain poorly understood in ancestral fishes such as sturgeons.

Fulton’s condition factor (K) represents standardized body mass by total length, and K implies condition of individuals (Nash *et al*., 2006). Many studies have reported that K is strongly influenced by extrinsic factors such as seasonality, food availability and geography (Sutton *et al*., 2000; Leclercq *et al*., 2010). Energy density (ED) is a unit of energy per volume or mass in animals (often expressed in J·g^−1^), which directly shows the status of energy reserves in an individual. ED is known to change with geographical distribution (Schultz and Conover, 1997) and developmental stages (Wuenschel *et al*., 2006). Specifically, energy allocation towards somatic growth and energy reserve during the first year of life is critical prior to winter, particularly in northern fishes where food resources become scarce and endogenous stores must be mobilized to sustain life (Post and Parkinson, 2001). Previous studies have shown that individuals with higher energy stores are more likely to survive prolonged winter conditions (Biro *et al*., 2004; Houston *et al*., 2014; Deslauriers *et al*., 2018).

**Figure 1:**
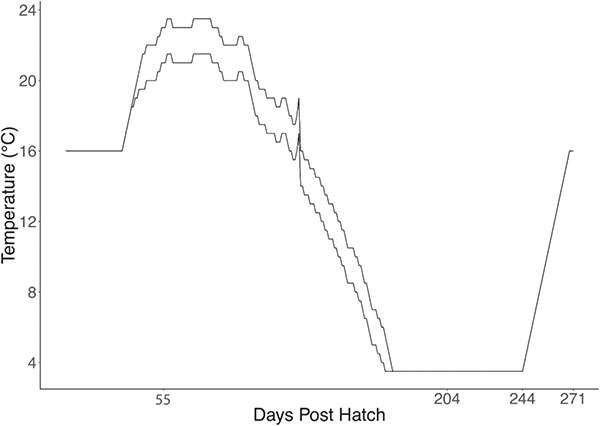
Temperature regimes used in this study. Days post hatch (dph) on the X-axis indicate when sampling occurred or there was a change in environmental parameters. ART denotes ambient river temperature. Two lines represent two different temperature regimes: (i) upper line: ART+2°C and (ii) lower line: ART. Dissolved oxygen remained constant within each experimental treatment. At 204 dph, food was deprived for 40 days following which temperature increased by 0.5°C per day. Temperature profiles used in this study were a reflection of natural temperature profiles collected from 2013 to 2016 at Slave Falls, on the Winnipeg River, MB, Canada. The average daily temperature was rounded to the nearest 0.5°C.

Lake Sturgeon, *Acipenser fulvescens*, Rafinesque 1817, is a large cartilaginous, benthic fish. They are native to North America and complete their full life history in freshwater. Lake Sturgeon were once widely distributed throughout North America (Peterson *et al*., 2007); however, they are now recognized as an endangered or threatened species across most of their natural range due to a variety of factors such as over-harvesting, hydroelectric dams and pollution. Despite conservation efforts for many decades, poor success has often been observed due in part to low recruitment rates of stocked fish. Specifically, a previous study showed that age-0 stocked Lake Sturgeon showed a relative recruitment rate 17.7 times lower than those stocked at age-1 (McDougall *et al*., 2014), which implies that phenotypes of fish produced from the hatchery may not be best suited to survive the first winter of life. Thus, it is necessary to understand how abiotic factors (e.g. temperature and DO) might shape phenotypic development in age-0 Lake sturgeon promoting increased survival rates during the first winter.

In this study, we examined how development of metabolic phenotypes in age-0 Lake Sturgeon was dependent on early rearing environments. Specifically, we were interested in variable temperature profiles, DO and substrate. We chose ambient river temperature (ART) and ART+2°C to represent natural temperature profiles that age-0 Lake Sturgeon may experience in Manitoba, Canada, and 80% of air-saturation DO was chosen as mild hypoxia is often observed in aquaculture due to high biomass density, loss of flow or in nature due to eutrophication (Diaz and Breitburg, 2009). Further, we included substrate based on the previous findings regarding its impacts on phenotypic development in sturgeon species (Gessner *et al*., 2009; McAdam, 2011; Zubair *et al*., 2012; Boucher *et al*., 2018; Yoon *et al*., 2018). Specifically, we tested the environmental matching hypothesis and predicted that fish raised in ART 100% DO with substrate would show the highest K, ED, MS and survival rate throughout the experiment. Furthermore, we predicted interactive effects of increased temperature and hypoxia would reduce forced maximum metabolic rate (FMR) and MS and that the presence of substrate would increase ED and K. Finally, assuming a fall stocking event, we examined how these phenotypes would influence differential survival rates during a simulated overwintering event.

## Materials and methods

### Animal husbandry and maintenance

Two females (28 ± 0.0 kg; mean ± S.E.) and three males (10.4 ± 1.21 kg; mean ± S.E.) were captured by gill net in the Nelson River, MB in May 2016. Upon fertilization, eggs were brought to Grand Rapids Fish Hatchery on 27 May 2016. Upon yolk absorption [8 days post-hatch (dph)] fish were fed to satiation three times a day with freshly hatched brine shrimp (Artemia International LLC, Texas, USA). Any uneaten brine shrimp was carefully removed by siphon after 15 min. At 20 dph, Lake Sturgeon larvae were transferred to the Animal Holding Facility at the University of Manitoba. Fish were distributed into 1 of 18 9L aquaria placed in a three-row multistressor unit (AquaBiotech, Quebec, Canada). Fish were acclimated to 16°C until 32 dph when temperature manipulation was initiated. ART was created based on the average daily temperature profile rounded up to the nearest 0.5°C recorded at Slave Falls on the Winnipeg River from 2013 to 2016 (Fig. 1), and 100% or 80% DO hereafter refers to treatments where tank water was maintained at 100% or 80% DO relative to air saturation in holding water regulated by injecting air or nitrogen gas. Rearing environments were maintained as one of the following: ART and 100% DO; ART+2°C and 80% DO; and ART+2°C and 100% DO. In each rearing environment, rearing tanks were evenly split between three tanks with substrate and the other three tanks without substrate. Each tank was supplied with dechlorinated City of Winnipeg tap water on a semi-recirculating system with a replacement rate of 10% per day for the whole unit, and temperature and DO in the treatment tanks were regulated within 0.2°C and 5%, respectively. Ammonia levels were observed to always be below 0.1 ppm across tanks (Ammonia Test Kit, Fluval, Quebec, Canada). Substrate consisted of sinking plastic pall rings with 25 mm diameter (Rashig USA INC, Texas, USA). At 22 dph, a mixture of ground bloodworm (Hikari USA, California, USA) and brine shrimp was fed to the fish, and the proportion of bloodworm was increased by 10% per day as the fish grew until they were on a diet of 100% bloodworm at 32 dph. At 32 dph, the pall rings were replaced with sand with sufficient amounts (2 cm height) to just cover the base of the tank and mimic the preferred habitat at this life stage in the wild (Benson *et al*., 2005). Photoperiod was set to correspond to the time of dawn and dusk at Pointe Du Bois on the Winnipeg River. Any mortalities were immediately removed, and daily survival in each treatment was calculated as the average survival across the three treatment tanks throughout the experimental period.

### Overwintering

A simulated overwintering event was performed with changes in temperature and light intensity similar to what has previously been described (Yoon *et al*., 2018) while DO remained constant within each treatment throughout the experimental period (Fig. 1). Temperature in all treatment tanks reached 3.5°C at 175 dph after which light intensity in all treatments was decreased gradually and remained at 10% of the initial brightness prior to the wintering period. At 204 dph, temperature and light intensity were maintained at 3.5°C and 10%, respectively, and food was deprived for 40 days to simulate an overwintering period. Following this period, temperature in all treatments was increased to 16°C at a rate of 0.5°C per day and light intensity was set to pre-winter levels. During this warming phase, fish in each treatment were fed bloodworm to satiation and were finally sampled within 4 days after all treatments reached 16°C.

**Table 1:** Mean ± SE mass (g) and total length (TL, mm) of Lake Sturgeon, *A. fulvescens*, raised under different rearing environments

**Treatment**	**Winter**	***n***	**Mass (g)**	**TL (mm)**
**ART 100% DO Sub**	Pre	15	3.39 ± 0.46	94 ± 5
	Post	29	3.12 ± 0.3	83 ± 3
**ART 100% DO NoS**	Pre	14	2.51 ± 0.19	86 ± 3
	Post	26	2.39 ± 0.19	78 ± 2
**ART+2°C 100% DO Sub**	Pre	nd	nd	nd
	Post	21	4.41 ± 0.66	95 ± 5
**ART+2°C 100% DO NoS**	Pre	6	3.58 ± 0.66	99 ± 6
	Post	30	3.01 ± 0.23	87 ± 2
**ART+2°C 80% DO NoS**	Pre	14	2.7 ± 0.30	90 ± 3
	Post	26	2.49 ± 0.19	80 ± 3^*^

Mass and length were assessed at 127 and 272 dph representing pre- and post-winter. ART denotes ambient river temperature, and ART+2 represents 2°C increased regime. DO represents the dissolved oxygen as percent air saturation. Sub and NoS indicate substrate and no substrate. While ‘^*^’ denotes a significant difference between pre- and post-winter values, nd denotes no data due to limited sample sizes. ART+2°C 80% DO Sub data were not collected due to limited sample sizes.

### Condition factor and Energy Density

At 127 and 271 dph, total length and body mass of fish were measured to the nearest 1 mm and 0.0001 g, respectively (Table 1). Due to higher mortality rates and limited numbers of fish reserved for the overwintering study, we were unable to sample from ART+2°C 100% DO with substrate at 127 dph and ART+2°C 80% DO with substrate at both 127 and 272 dph. From Table 1, K was calculated following the equation

$$\mathrm{K}=\frac{Weight\ (g)\times 100}{Total\ Length\ {(cm)}^3}.$$

Fish were sacrificed by immersion in an overdose of tricaine methanesulfonate (MS222; 250 mg·L^−1^; Syndel Laboratories, British Columbia, Canada) and length and wet mass were recorded. Carcasses were then placed in a drying oven and desiccated at 60°C for 48 h or until a constant mass was recorded. Dry to wet mass ratio was then used to predict the ED (J·g^−1^ of body mass) of individual fish using the model previously verified by Yoon *et al*. (2018).

### Metabolic rate

At approximately 122 and 269 dph, metabolic rate was assessed as whole body oxygen consumption rate ($\dot{M}{O}_2$) using intermittent flow respirometry as previously described in Yoon *et al*. (2018). The following parameters were used to assess $\dot{M}{O}_2$: a 360 s flushing period, 60 s wait period and 300 s measurement period (Loligo Systems, Viborg, Denmark). Flow rate was set to minimize stress on individuals, but sufficient to provide water exchange for measurement of $\dot{M}{O}_2$ on an intermittent basis. Respirometry chambers were surrounded by black curtains to avoid visual disturbance for each trial.

Individual fish were fasted for 12 h prior to measurement of $\dot{M}{O}_2$. Individuals were lightly anesthetized by immersion in 50 mg·L^−1^ of MS222 buffered with equal volumes of sodium bicarbonate for 10 s, and their mass and total length were recorded before being placed in the respirometry chamber. Our pre-experiment trials revealed that at least 6 h of acclimation was required to obtain SMR in age-0 Lake Sturgeon, so $\dot{M}{O}_2$ data were collected for the following 18 h to estimate SMR. Following this period, FMR was assessed using a standardized chase protocol where individuals were chased for 15 min with gentle prodding of the tail region with a plastic pipette. Fish were then immediately returned to the same respirometry chamber and oxygen consumption was measured for three measurement periods. Biological oxygen demand (BOD) was quantified by measuring oxygen consumption without fish for 15 min in each metabolic chamber before measurement of SMR and following measurement of FMR to correct for any increases in BOD during the time frame of the experiment. It is important to note that $\dot{M}{O}_2$ prior to the overwintering event was measured at 16°C and 18°C because these were corresponding temperatures at 122 dph, whereas $\dot{M}{O}_2$ following the overwintering event was measured at 16°C. All $\dot{M}{O}_2$ were conducted at 100% DO.

Analysis of metabolic rate data was conducted following the protocol used previously (Yoon *et al*. 2018). Slopes of declining oxygen concentration were collected for each measurement, and only coefficients of determination (*r^2^*) above 0.9 were used for analysis. Measured BODs in the beginning and end of each trial were then used to interpolate a linear slope over time to reflect BOD drifting, and all the data points were corrected by each corresponding BOD at each measurement time. The ${q}_{0.1}$ method (10th quantile) was used as previously described by Chabot *et al*. (2016) to estimate representative SMR values. FMR was determined by choosing the highest oxygen consumption rate among three measurements following the standardized chase protocol. MS was calculated by subtracting SMR from FMR.

### Critical Thermal Maximum

At 55 dph and 271 dph, CTmax was assessed to the nearest 0.01°C for individuals as described previously by Deslauriers *et al*. (2016) with slight modifications. It is important to note that due to the limited numbers of fish reserved for the overwintering study, we were unable assess CTmax at 127 dph (pre-winter). In brief, 48 small plastic containers with screen mesh were placed on a tray where the water temperature was increased by 2°C per hour. Initial water temperature was set to the same temperature of each rearing environment and was regulated by a thermostat (Fisher, Massachusetts, USA) while being well oxygenated. Once fish lost equilibrium and showed no response to gentle prodding, temperature was recorded in each container using a temperature probe equipped with a Witrox 4 Oxygen Meter (Loligo Systems, Viborg, Denmark). At the end of the trial fish were removed from their individual containers, measured and weighed before being euthanized by immersion in an overdose of MS222 (250 mg·L^−1^). All the experimental protocols were performed as described under animal use protocol F15-007 approved by the University of Manitoba Protocol Management Review Committee under the guidelines of the Canadian Council for Animal Care.

### Statistical analysis

A generalized linear mixed model (GLMM) was employed to analyse the data to account for the random effect of rearing tanks as previously described (Yoon *et al*., 2018). Temperature, DO and substrate are considered as discrete factor variables, and they are represented by T (ART to ART+2°C), DO (100% to 80%) and S (absence to presence), respectively. ${\alpha}_{ID}$ represents the intercept of random effects of rearing tanks, and $\varepsilon$ indicates residuals errors. The full model was written as follows:

$$\begin{align*} \hat{R}=&\ {\alpha}_0+{\beta}_t\cdotp T+{\beta}_{do}\cdotp DO+{\beta}_s\cdotp S+{\beta}_{t\ast s}\cdotp T\times S\\ &+{\beta}_{do\ast s}\cdotp DO\times S+{\alpha}_{ID}+\varepsilon, \end{align*}$$

where the response variable ($\hat{R}$) represents either condition factor (100 × g cm^−3^), ED (J·g^−1^), metabolic rate (mgO_2_ · kg^−1^·h^−1^) or CTmax (°C). The model was used to assess all variables independently at each sampling date across all treatments, and a likelihood ratio test was performed to test significance of each variable using the ‘anova’ function in the R package lme4 with backward elimination (Winter, 2013). When results indicated significance, *post hoc* comparisons were performed for each sampling date using the ‘glht’ function in the R package ‘multcomp’ (Hothorn *et al*., 2008; Allen *et al*., 2016; Yoon *et al*., 2018) with significance determined at alpha = 0.05 (see Tables S1 and S2 for the full results of *post hoc* analysis for data). Student’s *t*-test was performed to examine changes in each measured phenotype after the simulated overwintering event within treatments, and the results are summarized in Table S3. All statistical analyses were conducted in R (R Core Team, 2018).

## Results

Our results showed complex interactions between increased temperature, hypoxia and presence of substrate on the metabolic phenotypes in age-0 Lake Sturgeon.

### Growth and condition factor

**Figure 2:**
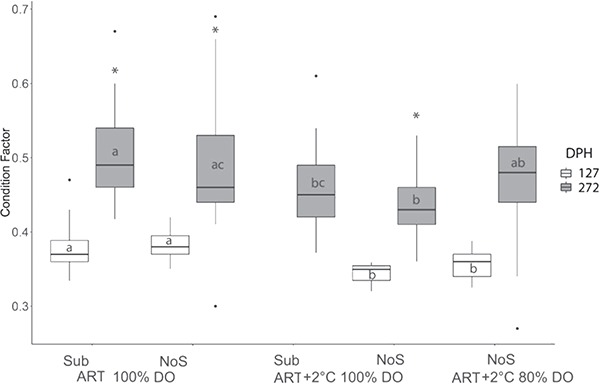
Condition factor of Lake Sturgeon, *A. fulvescens*, raised in different rearing environments DPH is days post-hatch for sampling. ART denotes ambient river temperature, and ART+2°C represents 2°C increased regime. DO represents the air saturation levels of dissolved oxygen. Sub and NoS indicate substrate and no substrate. Samples for ART+2°C 100% DO Sub at 127 dph and ART+2°C 80% DO Sub at both 127 and 272 dph were not collected due to limited sample sizes. Different letters represent significant difference between treatment at each sampling point. The ‘^*^’ denotes significant change after the simulated overwintering event.

Our data indicate that there was no significant effect of environmental variables on body mass or total length of age-0 Lake Sturgeon at 127 and 272 dph (Table 1); however, higher temperature reduced K at both sampling times (Table 2; *P* < 0.01). Specifically, at 127 dph, comparison of the individual treatments revealed that Lake Sturgeon raised in ART 100% DO showed a significantly higher K than those raised in ART+2°C 100% DO without substrate and ART+2°C 80% DO without substrate (Fig. 2; *P* < 0.05). At 272 dph, we saw a similar trend where Lake Sturgeon raised in ART 100% DO with substrate showed higher K than those raised in ART+2°C 100% DO. Within treatment analysis showed that K significantly increased in treatments of ART 100% DO and ART+2 °C 100% DO without substrate after the simulated overwintering event (Fig. 2).

**Table 2:** Analysis of condition factor (K) and energy density (ED) at 127 and 272 dph in Lake Sturgeon, *A. fulvescens*, raised in different rearing environments by generalized linear mixed model

**Measurement**	Dph	**Variables**	**Coefficient**	**Std. Error**	**χ** ^**2**^	***P***
**K**	**127**	α_0_	−0.97	0.02	na	na
		T (ART+2°C)	−0.10	0.03	9.514	**<0.001**
		DO (80%)	0.03	0.03	1.222	0.269
		S (Presence)	−0.00	0.02	0.005	0.944
	**272**	α_0_	72.68	7.59	na	na
		T (ART+2°C)	−29.41	10.84	9.669	**0.008**
		DO (80%)	28.85	10.71	5.727	**0.017**
		S (Presence)	15.37	10.89	3.174	0.205
		T × S	−2.16	15.60	0.019	0.891
**ED**	**127**	α_0_	2974.03	61.27	na	na
		T (ART+2°C)	−205.19	111.86	2.973	0.085
		DO (80%)	185.71	110.74	2.598	0.107
		S (Presence)	170.49	85.19	3.243	0.071
	**272**	α_0_	2538.59	74.05	na	na
		T (ART+2°C)	197.96	100.93	4.021	0.134
		DO (80%)	−302.3	99.67	6.731	**0.001**
		S (Presence)	−33.12	101.43	0.950	0.622
		T × S	−63.81	145.22	0.173	0.678

Results were obtained by likelihood ratio test with an elimination of each variable. Three variables are described in the table below: α_0_, intercept; T, temperature (ART+2°C compared to ART); DO, dissolved oxygen (80% of air saturation compared to 100%); S, substrate presence compared with absence, while ‘na’ means ‘not applicable’. χ^2^ denotes Chi-square value from the likelihood ratio test between the full model and reduced model while *P* is probability that is significant when bolded. Dph represents days post hatch for sampling. All the environmental variables were regarded as factor variables. Tanks were used as the random effect. Log and rank transformations were performed on the data of K at 127 and 272 dph, respectively, for the analysis.

### Energy Density

**Figure 3:**
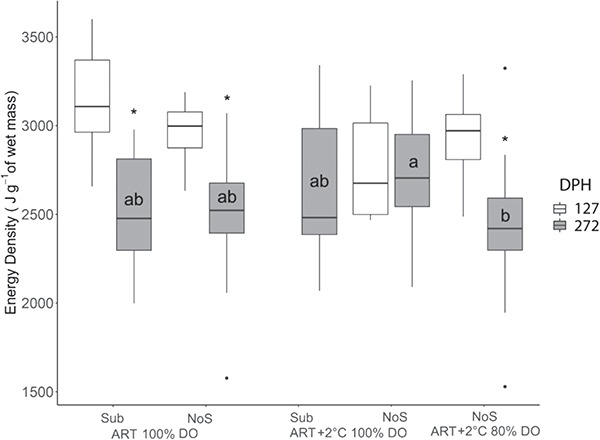
Energy density of Lake Sturgeon, *A. fulvescens*, raised in different rearing environments DPH is days post-hatch for sampling. ART denotes ambient river temperature, and ART+2 represents 2°C increased regime. DO represents the air saturation levels of dissolved oxygen. Sub and NoS indicate substrate and no substrate. Samples for ART+2°C 100% DO Sub at 127 dph and ART+2°C 80% DO Sub at both 127 and 272 dph were not collected due to limited sample sizes. Different letters represent significant difference between treatment at each sampling point. The ‘^*^’ denotes significant change after the simulated overwintering event.

There were no significant effects of environmental variables on ED at 127 dph. However, at 272 dph, DO had a significant effect on ED (Table 2; *P* = 0.001), and comparison of the individual treatments showed that Lake Sturgeon raised in ART+2°C 100% DO without substrate showed higher ED than those raised in ART+2°C 80% DO without substrate (Fig. 3). Within treatment analysis indicated that Lake Sturgeon raised in ART+2°C 100% DO without substrate did not change in ED after the winter, whereas Lake Sturgeon raised in ART 100% DO and ART+2°C 80% DO without substrate showed decreased ED (Fig. 3; *P* < 0.001)

**Figure 4:**
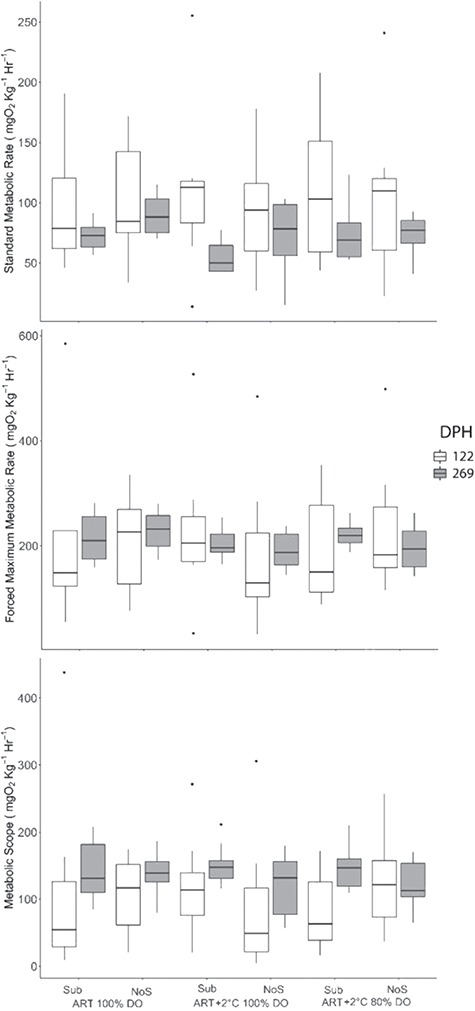
Metabolic rates and scopes of age-0 Lake Sturgeon, *A. fulvescens*, raised in different rearing environments Dph is approximate days post-hatch for sampling. ART denotes ambient river temperature, and ART+2 represents 2°C increased regime. DO represents the air saturation levels of dissolved oxygen. Sub and NoS indicate substrate and no substrate.

### Metabolic rate (SMR, FMR and MS)

Interestingly, our results indicated no significant effects of environmental variables on SMR, FMR and MS between treatments at each sampling point (Fig. 4).

### CTmax

**Figure 5:**
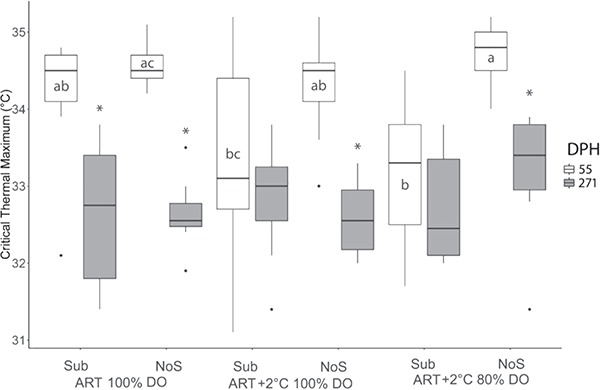
Critical thermal maximum of age-0 Lake Sturgeon, *A. fulvescens*, raised in different rearing environments DPH is days post-hatch for sampling. ART denotes ambient river temperature, and ART+2 represents 2°C increased regime. DO represents the air saturation levels of dissolved oxygen. Sub and NoS indicate substrate and no substrate. Different letters represent significant difference between treatment at each sampling point. The ‘*’ denotes significant change after the simulated overwintering event.

**Figure 6:**
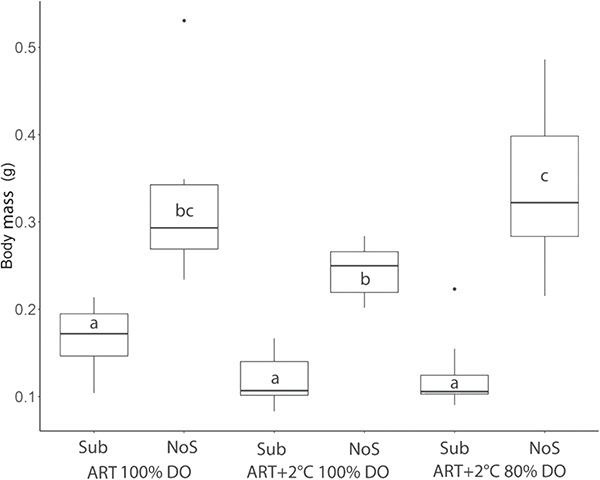
Body mass of age-0 Lake Sturgeon, *A. fulvescens*, used for CTmax experiment at 55 dph Fish were raised in different rearing environments. ART denotes ambient river temperature, and ART+2 represents 2°C increased regime. DO represents the air saturation of dissolved oxygen. Sub and NoS indicate substrate and no substrate. Different letters represent significant difference between treatments.

At 55 dph, only substrate had effects on CTmax (Table 3; *P* < 0.001); however, comparisons of the individual treatments showed that this was limited in the ART+2°C 80% DO treatment where presence of substrate significantly reduced CTmax (Fig. 5). Although not consistent with our findings from CTmax, at 55 dph we also observed significant differences in body mass by treatment used for the CTmax experiment (Fig. 6). Within treatment analysis indicated that CTmax did not decrease after the winter in two treatments of ART+2°C 100% DO with substrate and ART+2°C 80% DO with substrate, whereas the remaining treatments showed significant decreases in CTmax post-winter (Fig. 5; *P* < 0.001).

### Survival rate

**Figure 7:**
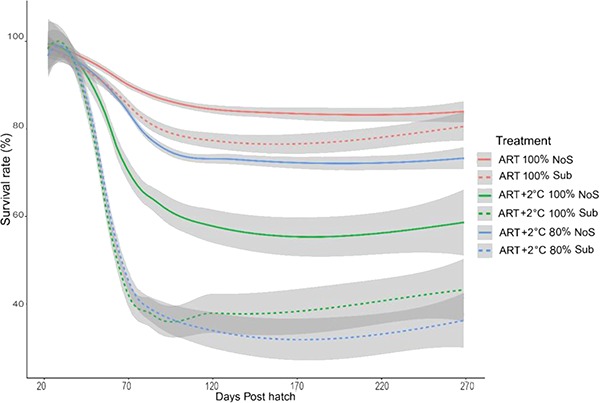
Survival rate of Lake Sturgeon, *A. fulvescens*, raised in different rearing environments Each treatment consists of three rearing tanks. Survival rate was calculated from the average at each date, and graph was generated with smooth function in ‘ggplot’ in R. ART denotes ambient river temperature, and ART+2 represents 2°C increased regime. DO represents the air saturation of dissolved oxygen. Sub and NoS indicate substrate and no substrate. Solid and dashed lines represent different treatments, and grey areas show the 95% confidence intervals.

Survival in all treatments decreased throughout development prior to the simulated overwintering (Fig. 7). The increased temperature appeared to have the greatest impairment on survival in all treatments, with hypoxia and presence of substrate also resulting in reduced survival rate prior to overwintering. Interestingly, no mortalities were observed across treatments during the simulated winter period.

## Discussion

### K and ED by environment

Early developing fish are known to be particularly sensitive to temperature as thermal stress can often result in developmental deformities and increases in oxidant stress during key life history phases (Werner *et al*., 2007; Simčič *et al*., 2015). We found a decrease in K for Lake Sturgeon reared under increased temperature at both pre- and post-winter sampling points, which suggests that the increased thermal regime for Lake Sturgeon in the present study was not optimal for energy assimilation. A similar trend was reported in White Sturgeon larvae that had a 7% reduction in condition factor when fish were reared at increased temperatures (14.5°C vs 17.5°C; Boucher *et al*., (2014)). In Shovelnose Sturgeon, *Scaphirhynchus platorynchus*, maximum feed conversion rate was found to be at 21.7°C, which was slightly lower than the optimal temperature for growth of 22.4°C. When environmental temperature exceeded 22.4°C for the same species, juveniles showed a reduced growth rate, feed efficiency and survival rate (Kappenman *et al*., 2009). Thus, fish reared in higher temperatures in our present study may experience lowered efficiency of energy assimilation and thus reduced K, which may have led to increased mortality rates.

Our data also showed that the inclusion of substrate did not improve K, which disagrees with previous studies (Gessner *et al*. 2009; Boucher *et al*., 2014; Yoon *et al*., 2018). Importantly, Boucher *et al*. (2014, 2018) removed substrate at the onset of the exogenous feeding stage, whereas in this study substrate remained either as pall rings or sand throughout the study period. Anecdotally, during the onset of exogenous feeding, brine shrimp often fell into the space between the substrate, which may have reduced foraging efficiency for fish raised over substrate. It has been reported that high mortality in the first year of life is not unusual in sturgeon (Caroffino *et al*., 2010) particularly at the onset of exogenous feeding (Gisbert *et al*., 2000). Our observation of more challenging access to food may explain lower survival rate in the substrate treatment groups during the larval development prior to our overwintering phase in the present study. Interestingly, in all but one of the treatments K was significantly higher post-winter at 272 dph, which is in disagreement with Yoon *et al*. (2018) where K was significantly lower post-winter regardless of treatment. It is well documented that fish that experience starvation replace lipid reserves and proteins with water (Martinez *et al*., 2003; Bar, 2014). Thus, it is possible that increases in water content during the overwintering event may have led to a perceived increase in K that was not supported by an increase in ED (see below).

**Table 3:** Analysis of Critical thermal maximum at 55 and 271 dph in Lake Sturgeon, *A. fulvescens*, raised in different rearing environments by generalized linear mixed model

**Dph**	**Variables**	**Coefficient**	**Std. Error**	**χ** ^**2**^	***P***
**55**	α_0_	34.67	4.19	na	na
	T (ART+2°C)	−5.00	5.93	4.007	0.135
	DO (80%)	10.33	5.93	3.582	0.167
	S (Presence)	−5.50	5.93	17.240	**<0.001**
	T × S	−6.00	8.38	0.510	0.475
	DO × S	−15.17	8.38	3.178	0.075
**271**	α_0_	32.64	0.24	na	na
	T (ART+2°C)	−0.05	0.34	0.417	0.812
	DO (80%)	0.60	0.34	3.171	0.205
	S (Presence)	−0.03	0.34	2.424	0.489
	T × S	0.26	0.48	0.302	0.583
	DO × S	−0.71	0.48	2.182	0.140

Results below were obtained by likelihood ratio test with an elimination of each variable. Dph represents days post hatch for sampling. Three variables are described in the table: α0, intercept; T, temperature (ART+2°C compared to ART°C); DO, dissolved oxygen (80% of air saturation presence compared with absence, while ‘na’ represents ‘not available’. χ2 denotes Chi-square value from the likelihood ratio test between the full model and reduced model while *P* is probability that is significant when bolded. All variables were regarded as factor variables, and replicate tanks were used as the random effect. Rank transformation was performed on the data at 55 dph to account for homoscedasticity in the data.

We saw a general decreasing trend of ED following the wintering event, which agrees with previous studies (Hurst, 2007; Deslauriers *et al*., 2018). It is thought that fish maximize their somatic growth as well as energy storage prior to winter such that they can survive when resources are likely to be scarce. Indeed, previous research showed that fish increase their lipid reserve from summer to fall (Booth and Keast, 1986; And and Kirkwood, 1995; Schultz and Conover, 1997). Specifically, energy allocation to lipid reserves may play an important role in overwintering as lipids were shown to be the critical energy source for fish exposed to periods of starvation (Byström *et al*., 2006). Because ED in fish is known to be positively correlated with lipid content (Anthony *et al*., 2000), our results emphasize that lipid reserves are important for surviving overwintering conditions.

Following the wintering event fish raised in increased temperature under hypoxia showed significantly lower ED than those raised under increased temperature regimes. It is unknown why ED was lower in this hypoxia group, but it is possible that metabolic adaptation to hypoxia at increased temperature may become detrimental during periods of starvation and reduced temperature.

### Metabolic rate by environment

Our data showed that increased rearing temperature had no impact on SMR, FMR and MS of Lake Sturgeon, which disagrees with Yoon *et al*. (2018). The discrepancy between these studies could be explained by the temperature and light regimes used in each study. While Yoon *et al*. (2018) used fixed rearing environments with temperature, the present study used a fluctuating temperature regime reflective of the natural environment. Cyclic changes in temperature are known to cause synchronism in many organisms across taxa such as cyanobacteria, algae, fungi and animals (Rensing and Ruoff, 2002; López-Olmeda, 2017). For example, reproductive cycles of adult fish are known to be regulated by annual photo-thermal cycles (Wang *et al*., 2010), and in Zebrafish, *Danio rerio*, changes in environmental temperature act as an important cue for the observed seasonal changes in swimming performance (Condon *et al*., 2010). Moreover, previous studies demonstrated some evidence of circadian rhythm in hormonal stress responses of Green Sturgeon, *Acipenser mediostris* (Lankford *et al*., 2003), metabolic rate in Lake Sturgeon (Svendsen *et al*., 2014) and migrating behaviours of adult Lake Sturgeon under full or new moon phases (Forsythe *et al*., 2012). Thus, natural environmental cues of temperature regimes and photoperiod used in this study may have triggered seasonal synchronism in age-0 Lake Sturgeon that induces subtle changes in regulation of energy balance throughout the experimental period.

The lack of change in SMR, FMR and MS between pre-and post-winter in the present study is also counter to previous studies (Mehner and Wieser, 1994; O’Connor *et al*., 2000; Yoon *et al.*, 2018) and may be explained by available energy. Deslauriers *et al*. (2018) demonstrated the critical level of endogenous energy reserves in age-0 Lake Sturgeon (~2000 J·g^−1^) to survive 40 days starvation at 1°C. Given that we found zero mortality during the wintering period our data may imply that fish did not reach the critical threshold to initiate metabolic changes. Further, ED following the overwintering event in this study was higher than the reported threshold of ED for survival.

In the present study 80% of air-saturation DO did not compromise aerobic metabolism in Lake Sturgeon and this is supported by Svendsen *et al*. (2014), but contrary to Yoon *et al*. (2018) who demonstrated that Lake Sturgeon raised at 80% of air-saturation DO showed lower survival and reduced aerobic capacity during a simulated overwintering event. It remains undetermined why results from these studies differ, but it is important to note that the Lake Sturgeon used in this study were from the Nelson River while Lake Sturgeon in the previous study came from the Winnipeg River. Interestingly, intraspecific variation in SMR has been explained by geographical adaptation of populations in isopods, *Porcellio laevis* (Lardies and Bozinovic, 2008), as well as family effects in Lake Sturgeon (Deslauriers *et al*. unpublished). Further, Stewart and Allen (2014) demonstrated intraspecific variation in thermal tolerance in Channel Catfish, *Ictalurus punctatus* and Hybrid Catfish, *Ictalurus furcatus*. Alternatively, the discrepancy may also be explained by the two different temperature regimes used. Natural changes of temperature may trigger seasonal changes in metabolic rate, which may regulate the effect of hypoxia on metabolic rate in this species.

### CTmax by environment

Our results showed that increased rearing temperature did not influence CTmax, which is in stark disagreement with previous studies where increases in acclimation temperature tend to increase CTmax (Beitinger and Bennett, 2000; Beitinger *et al*., 2000). However, several studies have reported that body mass may also have an important role in thermal tolerance in fish. For example, a positive relationship between body mass and CTmax was found in Shortnose Sturgeon, *Acipenser brevirostrum*, Shovelnose Sturgeon, *S. platorynchus* and Pallid Sturgeon, *Scaphirhynchus albus* (Zhang and Kieffer, 2014; Deslauriers *et al*., 2016). Therefore, the different body mass used in the CTmax experiments may in part explain our findings of reduced CTmax in the elevated temperature treatment group.

Many studies have reported that hypoxia could significantly reduce thermal tolerance of fish (Alabaster and Welcomme, 1962; Weatherley, 1970; Rutledge and Beitinger, 1989; Healy and Schulte, 2012). A positive correlation between CTmax and hypoxia tolerance was reported in Atlantic Salmon, *Salmo salar* (Anttila *et al*., 2013). Healy and Schulte (2012) reported that Killifish, *Fundulus heteroclitus*, exposed to 0.8 mgO_2_·L^−1^ for 4 weeks had a significant reduction in CTmax. However, our data showed that long-term exposure to hypoxia of 80% of air-saturation DO had no effect on CTmax of age-0 Lake Sturgeon.

### Survival rate

Increased temperature and hypoxia significantly reduced survival rate prior to the winter period. This is likely due to failure to appropriately respond to thermal and hypoxic stress during early development especially during the onset of exogenous feeding. This trend of decrease in survival is similar to Boucher *et al*. (2014), who reported decreased survival rate during larval development at higher temperatures (17.5°C vs 13.5°C). Also, a previous study reported that increased temperature reduced survival in juvenile Lake Sturgeon (Wehrly, 1995). It remains unknown why the survival rates were lower in the treatments with substrate, but again it is possible that the limited food availability at the critical developmental stage could result in underdeveloped larvae, which can be vulnerable to hypoxic stressors.

## Applications for conservation aquaculture

Increased temperature reduced condition factor and significantly reduced survival rate throughout the early development of Lake Sturgeon. 80% of air-saturation DO did not impact aerobic metabolism of Lake Sturgeon, but longer-term exposure to 80% of air-saturation DO significantly reduced their ED. In addition, our results suggest that substrate should be used for developing larval sturgeon for a short period during yolk sac absorption prior to emergence, to enhance survival rate in the first year. Further research is needed to understand what trade-offs allowed those fish to survive in the long term.

## Supplementary Material

Dynamic_Environment_Supplementary_Tables_June_4_Yoon_et_al_2019_coz055

## References

[ref1] Alabaster JS, Welcomme RL (1962) Effect of concentration of dissolved oxygen on survival of trout and roach in lethal temperatures. Nature 194: 170.

[ref2] Allen JL, Chown SL, Janion-Scheepers C, Clusella-Trullas S (2016) Interactions between rates of temperature change and acclimation affect latitudinal patterns of warming tolerance. Conserv Physiol 4: doi:10.1093/conphys/cow053. 10.1093/conphys/cow053 PMC5142048 27933165

[ref3] And DG, Kirkwood RC (1995) Seasonal variation in growth, mortality and fat stores of roach and perch in Lough Neagh, Northern Ireland. J Fish Biol 47: 537–554.

[ref4] Anthony JA, Roby DD, Turco KR (2000) Lipid content and energy density of forage fishes from the northern Gulf of Alaska. J Exp Mar Biol Ecol 248: 53–78. 10764884 10.1016/s0022-0981(00)00159-3

[ref5] Anttila K, Dhillon RS, Boulding EG, Farrell AP, Glebe BD, Elliott JAK, Wolters WR, Schulte PM (2013) Variation in temperature tolerance among families of Atlantic salmon (*Salmo salar*) is associated with hypoxia tolerance, ventricle size and myoglobin level. J Exp Biol 216: 1183–1190. 23487268 10.1242/jeb.080556

[ref6] Araki H, Berejikian BA, Ford MJ, Blouin MS (2008) Fitness of hatchery-reared salmonids in the wild. Evol Appl 1: 342–355. 25567636 10.1111/j.1752-4571.2008.00026.x PMC3352433

[ref7] Baker DW, Wood AM, Kieffer JD (2005) Juvenile Atlantic and Shortnose Sturgeons (Family: *Acipenseridae*) have different hematological responses to acute environmental hypoxia. Physiol Biochem Zool 78: 916–925. 16228931 10.1086/432860

[ref8] Bar N (2014) Physiological and hormonal changes during prolonged starvation in fish. Can J Fish Aquat Sci 71: 1447–1458.

[ref9] Bates LC, Boucher MA, Shrimpton JM (2014) Effect of temperature and substrate on whole body cortisol and size of larval white sturgeon (*Acipenser transmontanus* Richardson, 1836). J Appl Ichthyol 30: 1259–1263.

[ref10] Behrens JW, Steffensen JF (2007) The effect of hypoxia on behavioural and physiological aspects of lesser sandeel, *Ammodytes tobianus* (Linnaeus, 1785). Mar Biol 150: 1365–1377.

[ref11] Beitinger TL, Bennett WA (2000) Quantification of the role of acclimation temperature in temperature tolerance of fishes. Environ Biol Fishes 58: 277–288.

[ref12] Beitinger TL, Bennett WA, McCauley RW (2000) Temperature tolerances of North American freshwater fishes exposed to dynamic changes in temperature. Environ Biol Fishes 58: 237–275.

[ref13] Benson AC, Sutton TM, Elliott RF, Meronek TG (2005) Seasonal movement patterns and habitat preferences of age-0 Lake Sturgeon in the lower Peshtigo River, Wisconsin. Trans Am Fish Soc 134: 1400–1409.

[ref14] Biro PA, Morton AE, Post JR, Parkinson EA (2004) Over-winter lipid depletion and mortality of age-0 rainbow trout (*Oncorhynchus mykiss*). Can J Fish Aquat Sci 61: 1513–1519.

[ref15] Booth DJ, Keast JA (1986) Growth energy partitioning by juvenile bluegill sunfish, *Lepomis macrochirus* Rafinesque. J Fish Biol 28: 37–45.

[ref16a] Boucher MA, McAdam SO, Shrimpton JM (2014) The effect of temperature and substrate on the growth, development and survival of larval white sturgeon. *Aquaculture* 430: 139–148.

[ref16] Boucher MA, Baker DW, Brauner CJ, Shrimpton JM (2018) The effect of substrate rearing on growth, aerobic scope and physiology of larval white sturgeon *Acipenser transmontanus*. J Fish Biol 92: 1731–1746. 29691861 10.1111/jfb.13611

[ref17] Burton T, Killen SS, Armstrong JD, Metcalfe NB (2011) What causes intraspecific variation in resting metabolic rate and what are its ecological consequences? Proc Biol Sci 278: 3465–3473. 21957133 10.1098/rspb.2011.1778 PMC3189380

[ref18] Byström P, Andersson J, Kiessling A, Eriksson LO (2006) Size and temperature dependent foraging capacities and metabolism: consequences for winter starvation mortality in fish. Oikos 115: 43–52.

[ref19] Caroffino DC, Sutton TM, Elliott RF, Donofrio MC (2010) Predation on early life stages of Lake Sturgeon in the Peshtigo River, Wisconsin. Trans Am Fish Soc 139: 1846–1856.

[ref19a] Chabot D, Steffensen JF, Farrell AP (2016) The determination of standard metabolic rate in fishes. J Fish Biol 88: 81–121. 26768973 10.1111/jfb.12845

[ref20] Chivers DP, Ferrari MCO (2013) Tadpole antipredator responses change over time: what is the role of learning and generalization? Behav Ecol 24: 1114–1121.

[ref21] Clarke A, Johnston NM (1999) Scaling of metabolic rate with body mass and temperature in teleost fish. J Anim Ecol 68: 893–905. 10.1111/j.1365-2656.2010.01672.x 20180875

[ref22] Condon CH, Chenoweth SF, Wilson RS (2010) Zebrafish take their cue from temperature but not photoperiod for the seasonal plasticity of thermal performance. J Exp Biol 213: 3705–3709. 20952619 10.1242/jeb.046979

[ref23] Deslauriers D, Heironimus L, Chipps SR (2016) Lethal thermal maxima for age-0 Pallid and Shovelnose Sturgeon: implications for shallow water habitat restoration. River Res Appl 30: 1872–1878.

[ref24] Deslauriers D, Yoon GR, Earhart ML, Long C, Klassen CN, Gary Anderson W (2018) Over-wintering physiology of age-0 lake sturgeon (*Acipenser fulvescens*) and its implications for conservation stocking programs. Environ Biol Fishes 101: 623–637.

[ref25] Diaz RJ, Breitburg DL (2009) Chapter 1 the hypoxic environment. In Richards JG, Farrell AP, Brauner CJ, , Hypoxia, Ed 1 Fish Physiology Academic Press, New York.

[ref26] Dupont-Prinet A, Vagner M, Chabot D, Audet C, MacLatchey D (2013) Impact of hypoxia on the metabolism of Greenland halibut (*Reinhardtius hippoglossoides*). Can J Fish Aquat Sci 70: 461–469.

[ref27] Farrell AP, Richards JG (2009) Chapter 11 defining hypoxia: an integrative synthesis of the responses of fish to hypoxia. In Richards JG, Farrell AP, Brauner CJ, , Hypoxia, Ed 1 Fish Physiology Oxford University Press, New York.

[ref28] Fernandes MN, Barrionuevo WR, Rantin FT (1995) Effects of thermal stress on respiratory responses to hypoxia of a south American Prochilodontid fish, *Prochilodus scrofa*. J Fish Biol 46: 123–133.

[ref29] Forsythe PS, Scribner KT, Crossman JA (2012) Environmental and lunar cues are predictive of the timing of river entry and spawning-site arrival in lake sturgeon *Acipenser fulvescens*. J Fish Biol 35–53. 22747803 10.1111/j.1095-8649.2012.03308.x

[ref30] Frappell PB, Butler PJ (2004) Minimal metabolic rate, what it is, its usefulness, and its relationship to the evolution of endothermy: a brief synopsis. Physiol Biochem Zool 77: 865–868. 15674761 10.1086/425191

[ref31] Gessner J, Kamerichs CM, Kloas W, Wuertz S (2009) Behavioural and physiological responses in early life phases of Atlantic sturgeon (*Acipenser oxyrinchus* Mitchill 1815) towards different substrates. J Appl Ichthyol 25: 83–90.

[ref32] Gisbert E, Williot P, Castelló-Orvay F (2000) Influence of egg size on growth and survival of early stages of Siberian sturgeon (*Acipenser baeri*) under small scale hatchery conditions. Aquaculture 183: 83–94.

[ref33] Healy TM, Schulte PM (2012) Factors affecting plasticity in whole-organism thermal tolerance in common killifish (*Fundulus heteroclitus*). J Comp Physiol B 182: 49–62. 21698526 10.1007/s00360-011-0595-x

[ref34] Hendry AP et al. (2011) Evolutionary principles and their practical application. Evol Appl 4: 159–183. 25567966 10.1111/j.1752-4571.2010.00165.x PMC3352551

[ref35] Hobbs JPA, Mcdonald CA (2010) Increased seawater temperature and decreased dissolved oxygen triggers fish kill at the Cocos (Keeling) Islands, Indian Ocean. J Fish Biol 77: 1219–1229. 21039501 10.1111/j.1095-8649.2010.02726.x

[ref36] Hochachka PW, Somero GN (2001) Biochemical Adaptation: Mechanism and Process in Physiological Evolution, Ed 1 Oxford University Press, New York.

[ref37] Holt RE, Jørgensen C (2015) Climate change in fish: effects of respiratory constraints on optimal life history and behaviour. Biol Lett 11:20141032 doi:10.1098/rsbl.2014.1032. 25673000 PMC4360111

[ref38] Hothorn T, Bretz F, Westfall P (2008) Simultaneous inference in general parametric models. Biom J 50: 346–363. 18481363 10.1002/bimj.200810425

[ref39] Houston BE, Rooke AC, Brownscombe JW, Fox MG (2014) Overwinter survival, energy storage and reproductive allocation in the invasive round goby (*Neogobius melanostomus*) from a river system. Ecol Freshw Fish 23: 224–233.

[ref40] Hurst TP (2007) Causes and consequences of winter mortality in fishes. J Fish Biol 71: 315–345.

[ref41] Jo J, Kim Y (1999) Oxygen consumption of Far Eastern catfish, *Silurus asotus*, on the different water temperatures and photoperiods. J Korean Fish Biol 32(1): 56–61.

[ref42] Johnsson JI, Brockmark S, Näslund J (2014) Environmental effects on behavioural development consequences for fitness of captive-reared fishes in the wild. J Fish Biol 85: 1946–1971. 25469953 10.1111/jfb.12547

[ref43] Kappenman KM, Fraser WC, Toner M, Dean J, Webb MAH (2009) Effect of temperature on growth, condition, and survival of juvenile shovelnose sturgeon. Trans Am Fish Soc 138: 927–937.

[ref44] Kieffer JD (2000) Limits to exhaustive exercise in fish. Comp Biochem Physiol A Mol Integr Physiol 126: 161–179. 10938136 10.1016/s1095-6433(00)00202-6

[ref44a] Lankford SE, Adams TE, Cech JJ (2003) Time of day and water temperature modify the physiological stress response in green sturgeon. Acipenser medirostris. Comp Biochem Physiol - A Mol Integr Physiol 135: 291–302. 12781829 10.1016/s1095-6433(03)00075-8

[ref44b] Lardies MA, Bozinovic F, (2008) Genetic variation for plasticity in physiological and life-history traits among populations of an invasive species, the terrestrial isopod. Porcellio laevis. Evol Ecol Res 10: 747–762.

[ref45] Leclercq E, Taylor JF, Hunter D, Migaud H (2010) Body size dimorphism of sea-reared Atlantic salmon (*Salmo salar* L.): implications for the management of sexual maturation and harvest quality. Aquaculture 301: 47–56.

[ref46] Lindström J (1999) Early development and fitness in birds and mammals. Trends Ecol Evol 14: 343–348. 10441307 10.1016/s0169-5347(99)01639-0

[ref47] Lönnstedt OM, McCormick MI, Meekan MG, Ferrari MCO, Chivers DP (2012) Learn and live: predator experience and feeding history determines prey behaviour and survival. Proc Biol Sci 279: 2091–2098. 22237904 10.1098/rspb.2011.2516 PMC3321713

[ref48] López-Olmeda JF (2017) Nonphotic entrainment in fish. Comp Biochem Physiol A Mol Integr Physiol 203: 133–143. 27642096 10.1016/j.cbpa.2016.09.006

[ref49] Martinez M, Guderley H, Dutil J-D, Winger PD, He P, Walsh SJ (2003) Condition, prolonged swimming performance and muscle metabolic capacities of cod Gadus morhua. J Exp Biol 206: 503–511. 12502771 10.1242/jeb.00098

[ref50] McAdam SO (2011) Effects of substrate condition on habitat use and survival by white sturgeon (*Acipenser transmontanus*) larvae and potential implications for recruitment. Can J Fish Aquat Sci 68: 812–822.

[ref51] McBryan TL, Anttila K, Healy TM, Schulte PM (2013) Responses to temperature and hypoxia as interacting stressors in fish: implications for adaptation to environmental change. Integr Comp Biol 53: 648–659. 23784697 10.1093/icb/ict066

[ref52] McDougall CA, Pisiak DJ, Barth CC, Blanchard MA, Macdonell DS, Macdonald D (2014) Relative recruitment success of stocked age-1 vs age-0 lake sturgeon (Acipenser fulvescens Rafinesque, 1817) in the Nelson River, northern Canada. J Appl Ichthyol 30: 1451–1460.

[ref53] Mehner T, Wieser W (1994) Energetics and metabolic correlates of starvation in juvenile perch (*Perca fluviatilis*). J Fish Biol 45: 325–333.

[ref54] Monaghan P (2008) Early growth conditions, phenotypic development and environmental change. Philos Trans R Soc B Biol Sci 363: 1635–1645. 10.1098/rstb.2007.0011 PMC2606729 18048301

[ref55] Nash RDM, Valencia AH, Geffen AJ (2006) The origin of Fulton’s condition factor—setting the record straight. Fisheries 31: 236–238.

[ref56] Norin T, Clark TD (2016) Measurement and relevance of maximum metabolic rate in fishes. J Fish Biol 88: 122–151. 26586591 10.1111/jfb.12796

[ref57] O’Connor KI, Taylor AC, Metcalfe NB (2000) The stability of standard metabolic rate during a period of food deprivation in juvenile Atlantic salmon. J Fish Biol 57: 41–51.

[ref58] Peterson DL, Vecsei P, Jennings CA (2007) Ecology and biology of the lake sturgeon: a synthesis of current knowledge of a threatened north American *Acipenseridae*. Rev Fish Biol Fish 17: 59–76.

[ref59] Pörtner H-O (2010) Oxygen- and capacity-limitation of thermal tolerance: a matrix for integrating climate-related stressor effects in marine ecosystems. J Exp Biol 213: 881–893. 20190113 10.1242/jeb.037523

[ref60] Post JR, Parkinson EA (2001) Energy allocation strategy in young fish: allometry and survival. Ecology 82: 1040–1051.

[ref61] Poulsen SB, Jensen LF, Nielsen KS, Malte H, Aarestrup K, Svendsen JC (2011) Behaviour of rainbow trout Oncorhynchus mykiss presented with a choice of normoxia and stepwise progressive hypoxia. J Fish Biol 79: 969–979. 21967584 10.1111/j.1095-8649.2011.03069.x

[ref62] Rensing L, Ruoff P (2002) Temperature effect on entrainment, phase shifting, and amplitude of circadian clocks and its molecular bases. Chronobiol Int 19: 807–864. 12405549 10.1081/cbi-120014569

[ref63] Richards JG (2009) Chapter 10 metabolic and molecular responses of fish to hypoxia. In Richards JG, Farrell AP, Brauner CJ, , Hypoxia, Ed 1 Fish Physiology Oxford University Press, New York.

[ref64] Roberts LJ, Taylor J, Garcia De Leaniz C (2011) Environmental enrichment reduces maladaptive risk-taking behavior in salmon reared for conservation. Biol Conserv 144: 1972–1979.

[ref65] Roberts LJ, Taylor J, Gough PJ, Forman DW, Garcia de Leaniz C (2014) Silver spoons in the rough: can environmental enrichment improve survival of hatchery Atlantic salmon *Salmo salar* in the wild? J Fish Biol 85: 1972–1991. 25469954 10.1111/jfb.12544

[ref66] Rutledge CJ, Beitinger TL (1989) The effects of dissolved oxygen and aquatic surface respiration on the critical thermal maxima of three intermittent-stream fishes. Environ Biol Fishes 24: 137–143.

[ref67] Schulte PM, Healy TM, Fangue NA (2011) Thermal performance curves, phenotypic plasticity, and the time scales of temperature exposure. Integr Comp Biol 51: 691–702. 21841184 10.1093/icb/icr097

[ref68] Schultz ET, Conover DO (1997) Latitudinal differences in somatic energy storage: adaptive responses to seasonality in an estuarine fish (Atherinidae: *Menidia menidia*). Oecologia 109: 516–529. 28307335 10.1007/s004420050112

[ref69] Simčič T, Jesenšek D, Brancelj A (2015) Effects of increased temperature on metabolic activity and oxidative stress in the first life stages of marble trout (*Salmo marmoratus*). Fish Physiol Biochem 41: 1005–1014. 25935664 10.1007/s10695-015-0065-6

[ref70] Stewart HA, Allen PJ (2014) Critical thermal maxima of two geographic strains of channel and hybrid catfish. N Am J Aquac 76: 104–111.

[ref71] Sutton SG, Bult TP, Haedrich RL (2000) Relationships among fat weight, body weight, water weight, and condition factors in wild Atlantic Salmon Parr. Trans Am Fish Soc 129: 527–538.

[ref72] Svendsen JC, Genz J, Gary Anderson W, Stol JA, Watkinson DA, Enders EC (2014) Evidence of circadian rhythm, oxygen regulation capacity, metabolic repeatability and positive correlations between forced and spontaneous maximal metabolic rates in lake sturgeon *Acipenser fulvescens*. PLoS One 9: e94693 doi:10.1371/journal.pone.0094693. 24718688 PMC3981817

[ref73] Treberg JR, Killen SS, MacCormack TJ, Lamarre SG, Enders EC (2016) Estimates of metabolic rate and major constituents of metabolic demand in fishes under field conditions: methods, proxies, and new perspectives. Comp Biochem Physiol A Mol Integr Physiol 202: 10–22. 27139083 10.1016/j.cbpa.2016.04.022

[ref74] Wang N, Teletchea F, Kestemont P, Milla S, Fontaine P (2010) Photothermal control of the reproductive cycle in temperate fishes. Rev Aquac 2: 209–222.

[ref75] Weatherley AH (1970) Effects of superabundant oxygen on thermal tolerance of goldfish. Biol Bull 139: 229–238. 29332482 10.2307/1540139

[ref76] R Core Team, (2018) R: A language and environment for statistical computing. R Foundation for Statistical Computing, Fisheries Research Report Ann Arbor, Michigan. https://www.R-project.org/.

[ref77] Werner I, Linares-Casenave J, Van Eenennaam JP, Doroshov SI (2007) The effect of temperature stress on development and heat-shock protein expression in larval green sturgeon (*Acipenser mirostris*). Environ Biol Fishes 79: 191–200.

[ref78] Winter B (2013) Linear models and linear mixed effects models in R with linguistic applications, http://arxiv.org/pdf/1308.5499.pdf arXiv:1308.5499.

[ref79] Wuenschel MJ, Jugovich AR, Hare JA (2006) Estimating the energy density of fish: the importance of ontogeny. Trans Am Fish Soc 135: 379–385.

[ref80] Yoon GR, Deslauriers D, Enders EC, Treberg JR, Anderson WG (2018) Effects of temperature, dissolved oxygen and substrate on the development of metabolic phenotypes in age-0 Lake Sturgeon, *Acipenser fulvescens*: implications for overwintering survival. Can J Fish Aquat Sci. doi:10.1139/cjfas-2018-0399.

[ref81] Zhang Y, Kieffer JD (2014) Critical thermal maximum (CT _max_) and hematology of shortnose sturgeons (*Acipenser brevirostrum*) acclimated to three temperatures. Can J Zool 92: 215–221.

[ref82] Zubair SN, Peake SJ, Hare JF, Anderson WG (2012) The effect of temperature and substrate on the development of the cortisol stress response in the lake sturgeon, *Acipenser fulvescens*, Rafinesque (1817). Environ Biol Fishes 93: 577–587.

