# Supplementary material for: Influence of a dynamic rearing environment on development of metabolic phenotypes in age-0 Lake Sturgeon, Acipenser fulvescens
Source: Conserv Physiol. 2019 Oct 11;7(1):coz055. doi: 10.1093/conphys/coz055 (PMC6788496; doi:10.1093/conphys/coz055)
Supplement: Dynamic_Environment_Supplementary_Tables_June_4_Yoon_et_al_2019_coz055 [file dynamic_environment_supplementary_tables_june_4_yoon_et_al_2019_coz055.docx]

**< Supplementary Tables >**

Influence of a Dynamic Rearing Environment on Development of Metabolic Phenotypes in age-0 Lake Sturgeon, *Acipenser fulvescens*

*Gwangseok R. Yoon, David Deslauriers and W. Gary Anderson

*Author to whom correspondence should be addressed yoongs@myumanitoba.ca

Table S1. Summary of Tukey contrasts of condition factor (K) and energy density (ED) between treatments at 127 and 272 days post hatch (DPH).

| **Measurement** | **DPH** | **Tukey contrasts** | **Estimates** | **Std. Error** | **z value** | **P** |
| --- | --- | --- | --- | --- | --- | --- |
| **K** | 127 | ART 100 NoS - ART 100 Sub | 0.001647 | 0.023319 | 0.071 | 1.000 |
|  |  | ART+2 100 NoS - ART 100 Sub | -0.09757 | 0.030311 | -3.219 | **0.007** |
|  |  | ART+2 80 NoS - ART 100 Sub | -0.06351 | 0.023319 | -2.724 | **0.032** |
|  |  | ART+2 100 NoS - ART 100 NoS | -0.09922 | 0.030619 | -3.241 | **0.006** |
|  |  | ART+2 80 NoS - ART 100 NoS | -0.06516 | 0.023717 | -2.747 | **0.030** |
|  |  | ART+2 80 NoS - ART+2 100 NoS | 0.034062 | 0.030619 | 1.112 | 0.678 |
|  | **272** | ART 100 NoS - ART 100 Sub | -15.3727 | 10.8908 | -1.412 | 0.620 |
|  |  | ART+2 100 Sub - ART 100 Sub | -31.568 | 11.227 | -2.812 | **0.040** |
|  |  | ART+2 100 NoS - ART 100 Sub | -44.785 | 10.4685 | -4.278 | **<0.001** |
|  |  | ART+2 80 NoS - ART 100 Sub | -15.9382 | 10.7613 | -1.481 | 0.575 |
|  |  | ART+2 100 Sub - ART 100 NoS | -16.1953 | 11.57 | -1.4 | 0.628 |
|  |  | ART+2 100 NoS - ART 100 NoS | -29.4123 | 10.8356 | -2.714 | **0.052** |
|  |  | ART+2 80 NoS - ART 100 NoS | -0.5655 | 11.1187 | -0.051 | 1.000 |
|  |  | ART+2 100 NoS - ART+2 100 Sub | -13.217 | 11.1735 | -1.183 | 0.761 |
|  |  | ART+2 80 NoS - ART+2 100 Sub | 15.6298 | 11.4482 | 1.365 | 0.650 |
|  |  | ART+2 80 NoS - ART+2 100 NoS | 28.8467 | 10.7054 | 2.695 | 0.055 |
| **ED** | **272** | ART 100 NoS - ART 100 Sub | 33.12 | 101.43 | 0.326 | 0.998 |
|  |  | ART+2 100 Sub - ART 100 Sub | 134.15 | 104.41 | 1.285 | 0.700 |
|  |  | ART+2 100 NoS - ART 100 Sub | 231.07 | 97.5 | 2.37 | 0.123 |
|  |  | ART+2 80 NoS - ART 100 Sub | -71.23 | 100.17 | -0.711 | 0.954 |
|  |  | ART+2 100 Sub - ART 100 NoS | 101.03 | 107.62 | 0.939 | 0.882 |
|  |  | ART+2 100 NoS - ART 100 NoS | 197.96 | 100.93 | 1.961 | 0.285 |
|  |  | ART+2 80 NoS - ART 100 NoS | -104.34 | 103.51 | -1.008 | 0.852 |
|  |  | ART+2 100 NoS - ART+2 100 Sub | 96.93 | 103.93 | 0.933 | 0.884 |
|  |  | ART+2 80 NoS - ART+2 100 Sub | -205.37 | 106.44 | -1.929 | 0.301 |
|  |  | ART+2 80 NoS - ART+2 100 NoS | -302.3 | 99.67 | -3.033 | **0.020** |

Table S2. Summary of Tukey contrasts of critical thermal maximum (CTmax) between treatments at 55 DPH.

| Measurement | Tukey contrasts | Estimates | Std. Error | z value | p |
| --- | --- | --- | --- | --- | --- |
| **CTmax** | ART 100 NoS - ART 100 Sub | 5.5 | 5.928 | 0.928 | 0.940 |
|  | ART+2 100 Sub - ART 100 Sub | -11 | 5.928 | -1.856 | 0.430 |
|  | ART+2 100 NoS - ART 100 Sub | 0.5 | 5.928 | 0.084 | 1.000 |
|  | ART+2 80 Sub - ART 100 Sub | -15.833 | 5.928 | -2.671 | 0.081 |
|  | ART+2 80 NoS - ART 100 Sub | 10.833 | 5.928 | 1.828 | 0.449 |
|  | ART+2 100 Sub - ART 100 NoS | -16.5 | 5.928 | -2.783 | 0.060 |
|  | ART+2 100 NoS - ART 100 NoS | -5 | 5.928 | -0.843 | 0.959 |
|  | ART+2 80 Sub - ART 100 NoS | -21.333 | 5.928 | -3.599 | **0.004** |
|  | ART+2 80 NoS - ART 100 NoS | 5.333 | 5.928 | 0.9 | 0.947 |
|  | ART+2 100 NoS - ART+2 100 Sub | 11.5 | 5.928 | 1.94 | 0.378 |
|  | ART+2 80 Sub - ART+2 100 Sub | -4.833 | 5.928 | -0.815 | 0.965 |
|  | ART+2 80 NoS - ART+2 100 Sub | 21.833 | 5.928 | 3.683 | **0.003** |
|  | ART+2 80 Sub - ART+2 100 NoS | -16.333 | 5.928 | -2.755 | 0.065 |
|  | ART+2 80 NoS - ART+2 100 NoS | 10.333 | 5.928 | 1.743 | 0.503 |
|  | ART+2 80 NoS - ART+2 80 Sub | 26.667 | 5.928 | 4.499 | **< 0.001** |

Table S3. Student T-test of energy density (ED) between 127 and 272 DPH and critical thermal maximum (CTmax) between 55 and 272 DPH in Lake Sturgeon, *Acipenser fulvescens*, raised in different rearing environments.

| Measurement | Treatment | df | t | p |
| --- | --- | --- | --- | --- |
| **ED** | ART 100% DO Sub | 42 | 6.7246 | **<0.001** |
|  | ART 100% DO NoS | 38 | 4.978 | **<0.001** |
|  | ART+2°C 100% DO Sub | nd | nd | nd |
|  | ART+2°C 100% DO NoS | 34 | 0.20389 | 0.840 |
|  | ART+2°C 80% DO Sub | nd | nd | nd |
|  | ART+2°C 80% DO NoS | 38 | 5.1777 | **<0.001** |
| **K** | ART 100% DO Sub | 40.815 | -8.787 | **<0.001** |
|  | ART 100% DO NoS | 29.374 | -5.6995 | **<0.001** |
|  | ART+2°C 100% DO Sub | nd | nd | nd |
|  | ART+2°C 100% DO NoS | 31.267 | -7.3534 | **<0.001** |
|  | ART+2°C 80% DO Sub | nd | nd | nd |
|  | ART+2°C 80% DO NoS | 21.46 | -9.0985 | **<0.001** |
| **CTmax** | ART 100% DO Sub | 15 | 3.6499 | **<0.01** |
|  | ART 100% DO NoS | 15 | 10.258 | **<0.001** |
|  | ART+2°C 100% DO Sub | 15 | 0.72478 | 0.480 |
|  | ART+2°C 100% DO NoS | 15 | 6.0181 | **<0.001** |
|  | ART+2°C 80% DO Sub | 15 | 1.2806 | 0.220 |
|  | ART+2°C 80% DO NoS | 15 | 4.9592 | **<0.001** |
